# Predictive Modeling of Surface Wear in Mechanical Contacts under Lubricated and Non-Lubricated Conditions

**DOI:** 10.3390/s21041160

**Published:** 2021-02-07

**Authors:** Ali Rahman, Muhammad Khan, Aleem Mushtaq

**Affiliations:** 1 Department of Electrical Engineering and Information Technology, Technische Universität Darmstadt, 64283 Darmstadt, Germany; 2 School of Aerospace, Transport and Manufacturing, Cranfield University, Cranfield, Bedfordshire MK43 0AL, UK; Muhammad.A.Khan@cranfield.ac.uk; 3 Department of Electronics and Power Engineering, Pakistan Navy Engineering College, National University of Sciences and Technology, Karachi 75350, Pakistan; aleem@pnec.nust.edu.pk

**Keywords:** non-contact sensing, sensor measurement, Intelligent algorithms, lubrication, contact, wear, noise

## Abstract

The surface wear in mechanical contacts under running conditions is always a challenge to quantify. However, the inevitable relationship between the airborne noise and the surface wear can be used to predict the latter with good accuracy. In this paper, a predictive model has been derived to quantify surface wear by using airborne noise signals collected at a microphone. The noise was generated from a pin on disc setup on different dry and lubricated conditions. The collected signals were analyzed, and spectral features estimated from the measurements and regression models implemented in order to achieve an average wear prediction accuracy of within 1${mm}^{3}$.

## 1. Introduction

Mechanical structures and components generate airborne noise due to their contact mechanics even in normal operational conditions. The amplitude and the frequency of the noise have a direct relation with the surface wear of the contact zone [1]. As the material wears out due to contact, a rapid release of energy from localized sources within a material causes a noise to be generated, hence the two factors; noise and wear seem in relation with each other [2]. Hence, it is not wrong to say that the spectrum of the emitted noise can be evaluated for the health diagnosis of machine components; its wear and friction resistance, as confirmed from the literature [3,4,5].

The correlation between wear and generated noise is not a new phenomenon for machine health diagnosis. This has been established since the 1970s [6]. Studies indicate the effectiveness of analyzing contact based acoustic emissions in order to calculate the wear occurred in a component for various machine processes like sliding, cutting, milling, and tool manufacturing [1,7,8,9,10,11,12,13,14,15,16]. The spectrum of the obtained acoustic emissions is further processed to measure count rates, RMS voltage, and also FFT analysis [10,13]. The signal analysis provides the acoustic parameter, whereas a wear parameter is also measured (wear area, wear volume, degradation, coefficient of friction) in order to correlate the two parameters [17,18,19]. The tool wear monitoring has been a regular exercise for industrial applications [20].

For this purpose, recently, Kong et al. described the methods of online tool wear monitoring. They used Kernel principal component analysis to fuse the sensitive tool wear signals. They constructed a predictive model for tool wear based on support vector regression and correlated the fused signal and the actual tool wear. The results showed that the prediction accuracy was close to 100% [21]. Another study used Titanium cylindrical flat pins over a Silicon carbide abrasive disc under dry sliding conditions [22]. It used the Taguchi method to predict wear behavior against variable load, speed, sliding duration, and signal-to-noise ratio. The obtained results showed a good correlation of wear and acoustic emissions under variable operating conditions, and a predictive model based on linear regression equation was also developed. Contact based signals were also studied under the effect of lubrication in the past [23,24,25]. Recently, researchers showed the possibility of the trend modeling between acoustic emissions and the wear in terms of accumulated signal energy and changes in spectrum amplitude [9,25,26,27,28,29]. However, most of these efforts used a single pin/point contact sensor and analyzed the spectrum of noise with different speed, sliding distance, and temperature values.

An extensive research literature is available on signal processing techniques that are used for analyzing acoustic emissions extracted from a contact based sensor and further correlated with the possible surface wear parameters. Several regression techniques are used in developing these correlations. However, the predictive modeling of wear volume through a non-contact microphone, still requires comprehensive effort. A reliable quantification of surface wear volume would be a major breakthrough in the field of condition based monitoring and early fault detection. In this paper, a predictive model has been derived and used to quantify the surface wear volume in a surface contact by using airborne noise recorded by means of a non-contact microphone. Airborne noise was generated from the operation of a pin on disc setup. The setup was run on different loads with and without lubrication. The noise was analyzed in order to determine features to extract, which were then computed via Non-Parametric Spectral Estimation. These were then considered along with the nature of the data acquisition experiments in order to formulate an appropriate predictive model.

## 2. Method

### 2.1. Experimental Setup and Wear-Noise Generation Scheme

**Figure 1:**
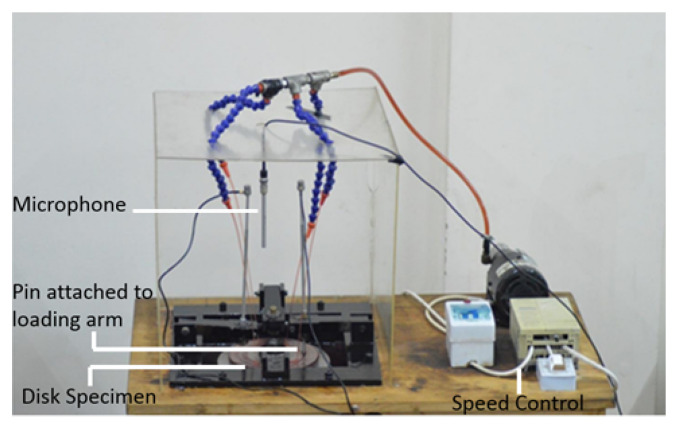
Pin on disc tribometer.

Authors have used the experimental rig as described in their recent work on noise and wear correlation [1]. In total, 37 experiments were performed on a pin on disc tribometer as shown in Figure 1 and considered for the statistical model development and validation.

The tribometer was composed of metallic arms to act as pin holders. A circular mild steel disc was mounted on a shaft and motor assembly that allowed the disc to rotate at a constant speed of 250 rpm. A high speed steel material pin was used and assembled with the help of the pin holder in a way so that it made a prefect mechanical contact with the circular disc as shown in Figure 1. The pin had a diameter of 5 mm and a contact diameter of 0.04 mm. Additional details about the pin’s material are listed in Table 1.

The pin tip at contact was made semi-circular in shape. It was assumed that the pin would not be worn out under sliding conditions at any time of experiments and hence the change in airborne noise would only be caused due the surface wear of the disc as it was made of a softer material. Threads of up to 10 mm in length were present on the other end of the pin, which were used to mount metallic weights and allowed to provide a tangential loading condition on the pin and disc contact during experiments. An oil pump was also used to provide lubrication at the contact and at a rate of 5 mL/s. The selected grade of lubricant was 10W-30 with a density of 877 kg/m${}^{3}$.

**Figure 2:**
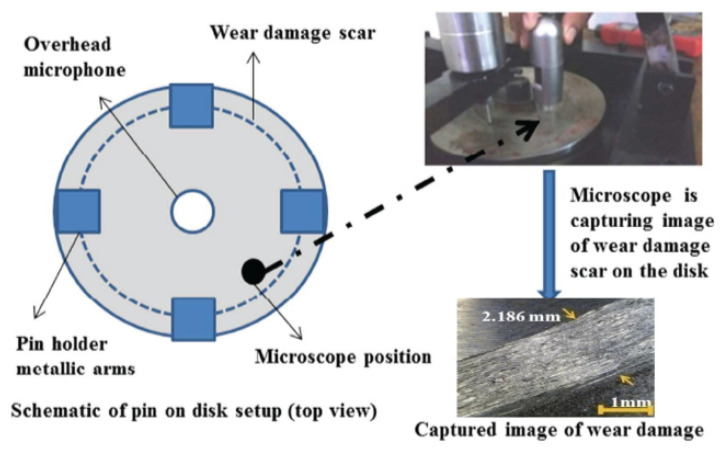
Portable microscope used to capture images of the disc scar (Image reused under STM Guidelines. Content rights are owned by and permission requests for further reuse are handled by SAGE Publishing, CA, USA) [1].

The noise was recorded by a GRAS 40PP free-field microphone (GRAS Sound & Vibration, Holte, Denmark). The frequency range of the selected microphone was 20 kHz, and it was able to record noises with an upper limit of 128 db. It was vertically placed at the center of the disc as shown in Figure 1. The raw noise signals were acquired by an NI 9234 data acquisition module (National Instruments, Austin, TX, USA) in terms of sound pressure (units pascal) and sampling rate of 25.6 kHz. Images of the wear scars on the disc were taken from six different positions, the same as it was performed in the previous work, with a portable microscope (Dino-Lite AM413T, AnMo Electronics Corporation, Hsinchu, Taiwan) at a magnification of 200× as shown in Figure 2 [1].

Using ASTM standard G99 (Standard Test Method for Wear Testing with a Pin-on-Disc Apparatus), disk volume loss *V* due to wear scar was calculated [17]. The wear scar sliding length measurements were measured from the captured images and used in ASTM standard formulation as provided in Equation (1):

(1) $$V=2\pi R\left[r^{2}\arcsin\left(\frac{d}{2r}\right)-\left(\frac{d}{4}\right)\sqrt{4r^{2}-d^{2}}\right]$$

where *R* = wear track radius, *d* = wear track width, *r* = pin end radius, and *V* is the volumetric wear, assuming no significant pin wear.

The load and lubrication specification breakdown for the 37 experiments is given in Table 2. The duration of each experiment was 6 min, with the wear on a disc measured after 30 s intervals, thus resulting in a total of 444 measurements.

### 2.2. Signal Analysis and Feature Extraction

Cutting processes tend to be stochastic [30] with generated noise consequently dependent upon the progression of the tool wear [31]. Hence, the stationarity of the collected data needs to be determined in order to decide the features extraction methodology. If the acquired signals turn out to be non-stationary, then Time-Frequency features would need to be computed; otherwise, frequency domain features would suffice [32]. To help with the analysis for stationarity, the mean and Autocorrelation Sequence (ACS) of one of the 30 s measurements was computed. The ACS of a process *X* can be defined as:

(2) $$R_{xx}\left[k\right]=E\left\{X\left[n\right]X\left[n+k\right]\right\}$$

where *n* is the time index, *k* the lag index, and *E* the expectation operator. Hence, the ACS is a measure of the correlation between two samples of the same stochastic process separated by a lag *k*.

**Figure 3:**
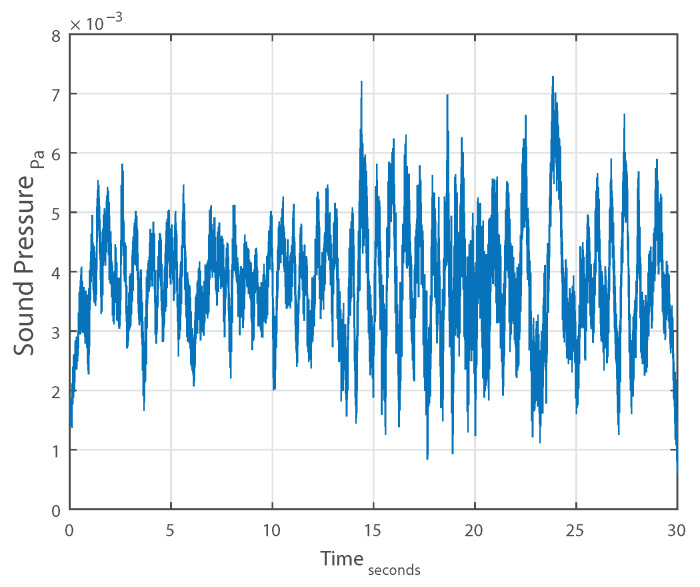
Moving average of a single measurement.

**Figure 4:**
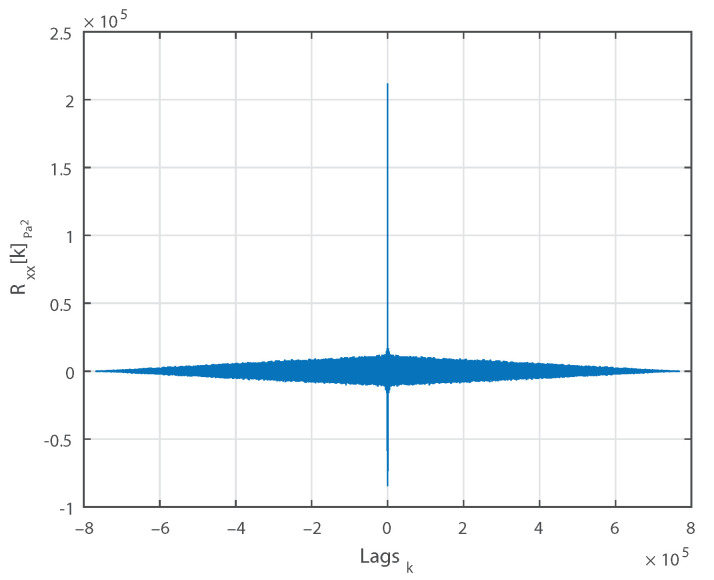
Autocorrelation sequence of a single 30 s long measurement.

**Figure 5:**
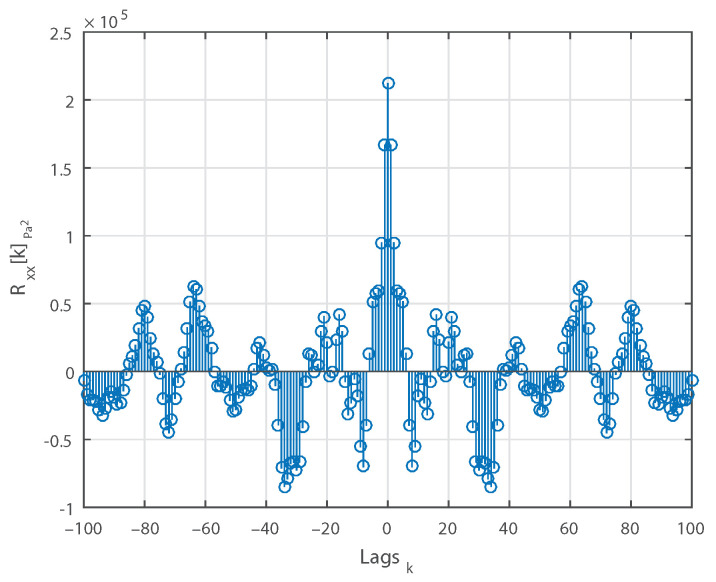
Autocorrelation sequence with lags limited to 100.

The evolution of the mean of a 30 s long measurement, computed using a moving average, is plotted against time in Figure 3. Figure 4 shows the computed ACS against the lag indexes for the same measurement. Figure 5 displays the same ACS with the lag index limited to |100|. Observing the three figures, we can conclude that:

1. The mean μ is relatively constant.
2. For k=0, $R_{xx}\left[k\right]$ is positive.
3. $R_{xx}\left[k\right]$ is an even function.
4. $R_{xx}\left[0\right]$ is $\max\{R_{xx}\left[k\right]\}$.
5. $R_{xx}\left[k\right]$ approaches $\mu^{2}$ as *k* increases.

**Figure 6:**
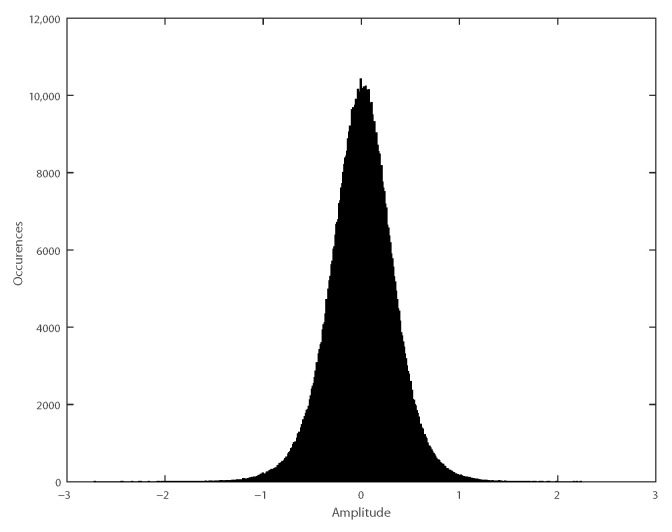
Distribution of measured signals’ samples.

Hence, the measurement can be classified as a wide-sense stationary (WSS) process [33]. Moreover, since the measured signal resembles a Gaussian distribution, as seen in Figure 6, this WSS process can be considered strict stationary (SS) as well [34].

Given the result of the stationarity analysis, the power spectral density (PSD) can be estimated. According to the Wiener–Khinchin theorem, the PSD is the squared discrete Fourier transform (DFT) of the ACS of each measurement signal [35]. The conventional periodogram estimator is considered analogous to this definition [36].

Since using the periodogram would have resulted in large fluctuations around the actual PSD due to the method’s inherent asymptotic noise [37], it is imperative to revert to techniques that reduce spectral variance.

Hence, the Welch method [38], which involves the segmentation of the signal being analyzed, and averaging of periodograms can be used. While segmentation reduces spectral resolution, the use of segment overlap mitigates this reduction when compared to the Bartlett method for the same number of segments [39]. The subsequent generation of redundant spectral information due to the use of overlapping is attenuated through the application of a windowing function, such as the Hamming window [40], over each segment. The Welch method for PSD estimation can be summarized in the following steps:

For periodograms k=1,…,K, a segment *k* is defined as:

(3) $$X_{k}\left(n\right)=X\left(n+\left(k-1\right)D\right)\;n=0,\ldots ,M-1$$

with *M* being the length of each segment and *D* controlling the degree of overlap. The number of periodograms involved, *K*, is dependent upon the previous two parameters and, *N*, the total length of the signal.

(4) $$K=\frac{N-M}{D}+1$$

Each periodogram can be defined as:

(5) $$I_{WX,WX}^{M}\left(e^{jw},k\right)=\frac{1}{MZ}{\left|\sum_{n=0}^{M-1}w\left(n\right)X_{k}\left(n\right)e^{-jwn}\right|}^{2}$$

where *Z* is the normalization factor considered due to the introduction of the Hamming window function w(n). By averaging this ensemble of periodograms, the Welch estimator is defined as:

(6) $${\tilde{C}}_{XX}\left(e^{jw}\right)=\frac{1}{K}\sum_{k=1}^{K}I_{WX,WX}^{M}\left(e^{jw},k\right)$$

For each measurement of duration 30 s, a Welch estimator with M=1280 was used along with an overlap of 50%. Since sampling frequency used was 25.6 kHz, N=768,000. Consequently, the PSD estimates ${\tilde{C}}_{XX}\left(e^{jw}\right)$ have a spectral resolution of 20 Hz. A lower window length wasn’t set in order to prevent spectral smearing and higher bias.

**Figure 7:**
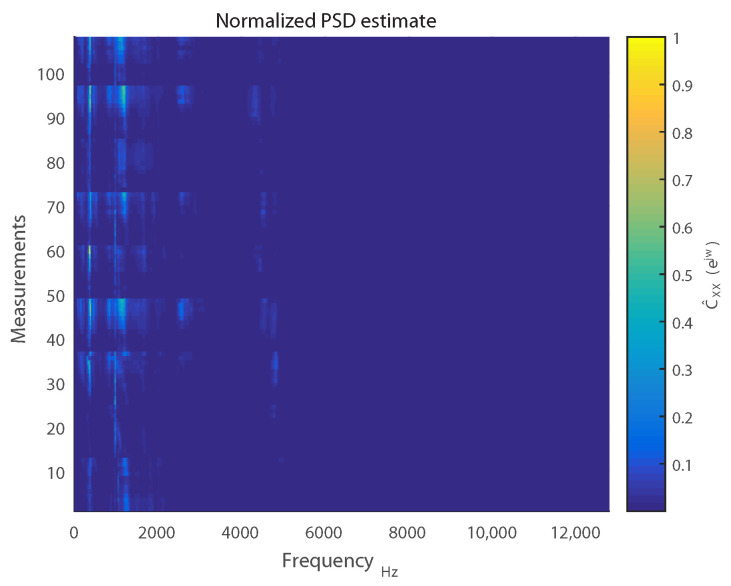
Normalized PSD estimates.

For the measurements we recorded, it was observed that the frequency ranges of interest were 0≤f≤ 3 kHz and 4≤f≤ 5 kHz. This can be visualized in Figure 7, which displays ${\tilde{C}}_{XX}\left(e^{jw}\right)$ for 108 measurements of 30 s each. For the sake of visual brevity, only measurements corresponding to absence of load and lubrication have been included.

The identification of these spectral regions of interest led to selection of the PSD estimates of 200 discrete frequency bins as features for our eventual surface wear prediction model.

### 2.3. Prediction Model Formulation

Since volumetric loss of one disc was measured over a span of 6 min in intervals of 30 s, they are cyclically cumulative, and the task of surface wear prediction can be modeled as a time series forecasting problem with a time horizon [41] of depth 12.

**Figure 8:**
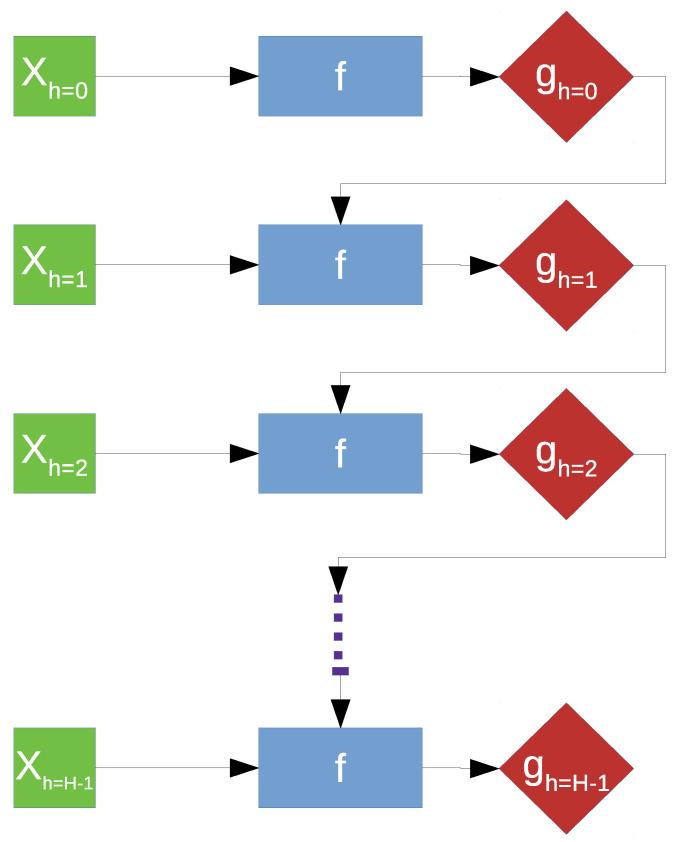
Recursive approach to time series forecasting.

For subsequent discussion, let *h* denote the time horizon, with $y_{h}$ and $X_{h}$ corresponding to the surface wear and spectral features of the measurement at horizon *h*. The model for predicting surface wear estimates $g_{h}$ can be expressed as *f*. A common solution for a forecasting problem is the recursive approach, which involves the prediction being fed back to the model, hence the name, as part of the input for prediction of the target belonging to the next time horizon [42]. However, this approach, illustrated in Figure 8, is extremely sensitive to prediction error due to error propagation [43].

**Figure 9:**
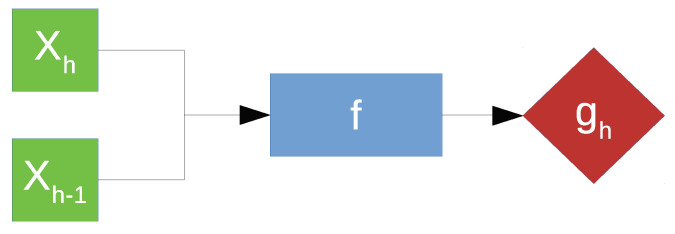
Proposed approach.

However, the time series aspect of the problem could not be ignored since the quantity to be predicted is the cumulative surface wear, with each 30 s wear process dependent on the previous one. Hence, we implemented time-embeddings using a sliding window approach for our spectral feature s [44]. Consequently, we arrive at a regression problem defined in Figure 9.

A sliding window, consisting of inputs belonging to the current time horizon *h* and the previous one h−1, is used as the new inputs which will be used to predict the cumulative surface wear at horizon *h*. In other words, we have set up a high dimensional regression problem with 400 spectral features as input for every cumulative surface wear output.

### 2.4. Choice of Regression Models

As can be discerned in Figure 7, the spectral features exhibit collinearity due to similar PSD estimates between some neighboring discrete frequency bins. Accordingly, a least-squares (LS) linear regression based model will not be optimal for prediction [45]. An optimal solution will seek to constrict the effects of collinearity between these features by attenuating the weights assigned to them during regression [46]. One method would be to implement a subset selection of features. However, this approach exhibits high variance due to the binary process of picking or dropping a feature entirely. Consequently, this would result in negligible improvement in prediction performance [46].

Ridge regression [47] is the alternate approach that can be adopted. It is a technique involving the shrinking of feature weights by imposing a penalty on their sizes. For a regression model defined by Equation (7), where X is an input matrix of features, y is the vector of output targets, and w the vector of weights assigned to features:

(7) y=wX

The LS linear regression solution would be the set of weights that minimize the sum of squared errors between the targets and predictions, as defined in Equation (8):

(8) $$\hat{w}=\underset{w}{\arg\min}{\left(y-w^{T}X\right)}^{T}\left(y-w^{T}X\right)$$

Comparatively, the optimization function for ridge regression includes the additional β parameter in order to penalize the size of weights as discussed previously. Hence, converging to the solution for Equation (9), requires reduction in the magnitudes of feature weights.

(9) $$\hat{w}=\underset{w}{\arg\min}\left({\left(y-w^{T}X\right)}^{T}\left(y-w^{T}X\right)+\beta w^{T}w\right)$$

While this causes an estimation bias, it decreases prediction error due to a reduction in variance [48]. The superiority of this method over feature subset selection has been proven in the work of Frank and Friedman (1993) [49].

Both LS linear regression and ridge regression suffer from a common drawback, the underlying linearity assumption. Accordingly, they offer poor and highly biased predictions when the relationship between features and the target is more complex. To overcome this issue, we will be moving on from the ridge regression method to Kernel Ridge Regression (KRR) [50], through which our spectral features will be mapped into a different dimensional space using a nonlinear kernel.

The Chi-Squared ($\chi^{2}$) kernel [51], defined in Equation (10), is used. The kernel utilizes the $\chi^{2}$ distance between two vectors *a* and *b* in order to generate a new feature vector, with γ controlling the variance of the kernel. This parameter allows us to improve the generalization of our regression model and reduce overfitting:

(10) $$\kappa \left(a,b\right)=\exp\left(-\gamma \sum_{i}\frac{{\left(a\left[i\right]-b\left[i\right]\right)}^{2}}{a[i]+b[i]}\right)$$

Our choice of kernel function is influenced by the assumption that our spectral features consisting of PSD estimates are similar to sparse histogram features for which the $\chi^{2}$ kernel performs well [52].

## 3. Results and Discussion

We used the dataset of our collected measurements to train and validate the following regression models, using Scikit-learn [53]:

- LS Linear Regression
- Ridge Regression
- $\chi^{2}$ KRR

The dataset was randomly sorted and partitioned such that 404 measurements were used as the training set while the remaining 40 were kept for validation. The training set could not be reduced further; otherwise, the quantity of observations available would be lower than the number of features, which would bar convergence to a unique solution for LS linear regression [54].

Two metrics were used in order to evaluate the performance of the regression models: the R-squared ($R^{2}$) and the Mean Absolute Error (MAE). The former determines the model’s goodness-of-fit and how well its predictions map towards the target variables [55], while the latter is a statistic denoting the mean of the error magnitudes between the target values and the model’s predictions [56]. In our context, the MAE represents the mean disparity, in mm${}^{3}$, between actual and predicted surface wears.

The performance of the models is summarized in Table 3.

These results were achieved with the following model parameters:

- β: 0.15
- γ: 0.1

**Figure 10:**
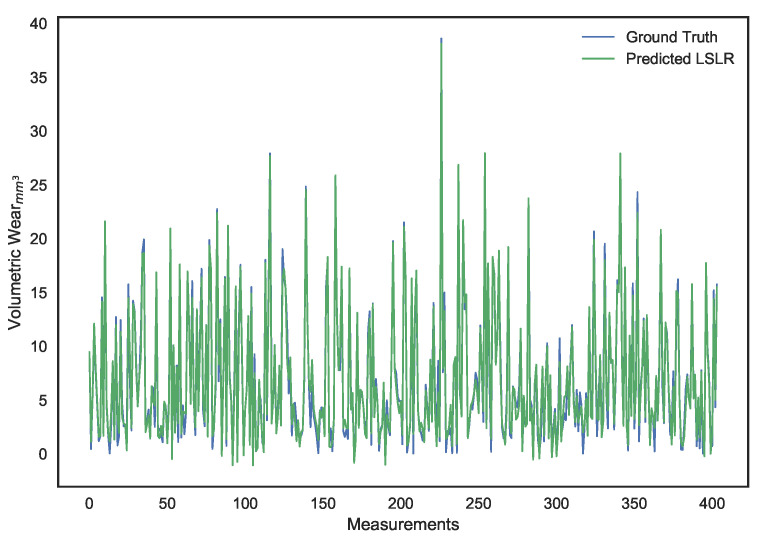
Surface wear prediction on the training set using least squares linear regression.

**Figure 11:**
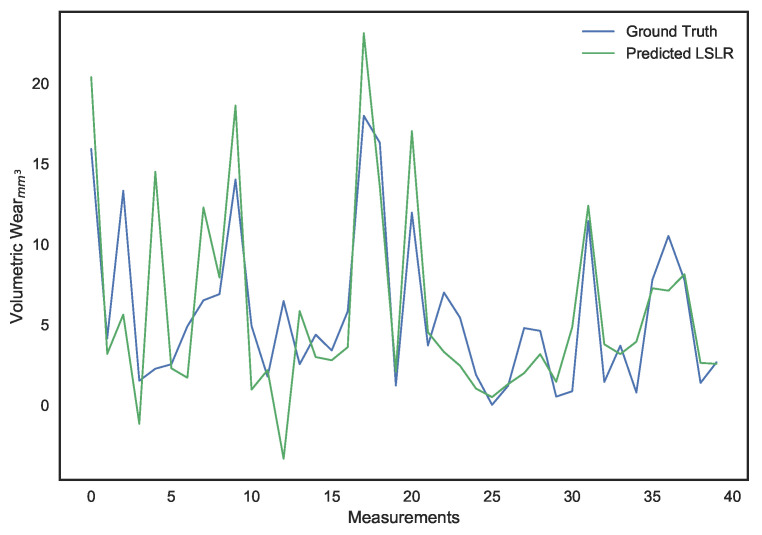
Surface wear prediction on the validation set using least squares Linear Regression.

**Figure 12:**
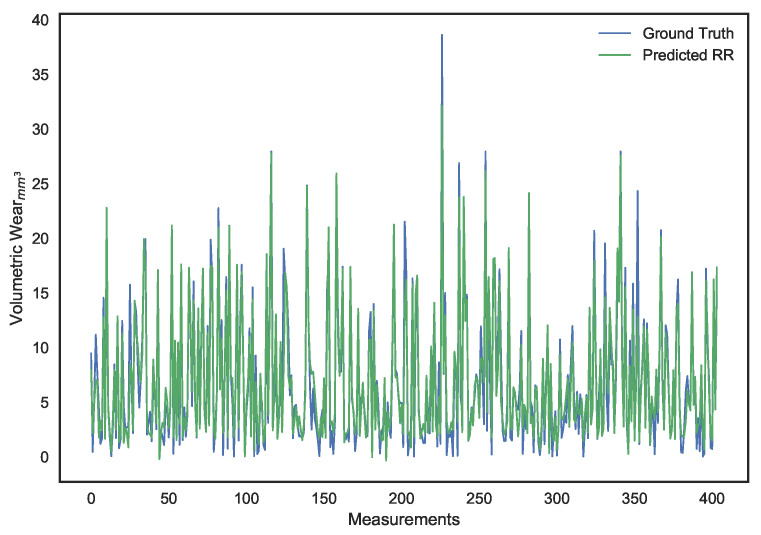
Surface wear prediction on the training set using ridge regression.

**Figure 13:**
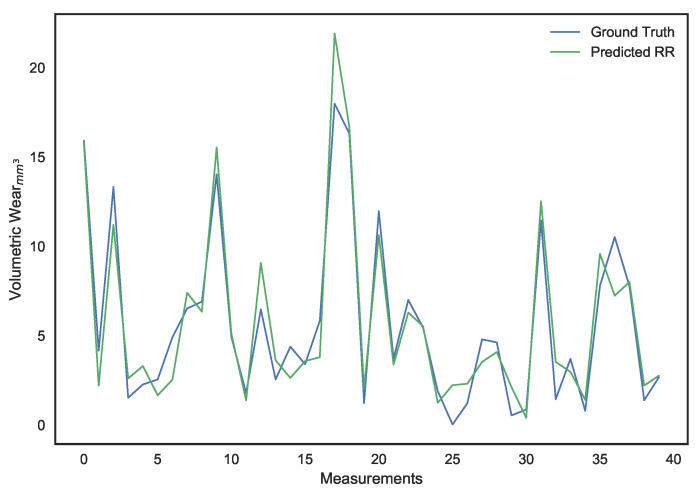
Surface wear prediction on the validation set using ridge regression.

**Figure 14:**
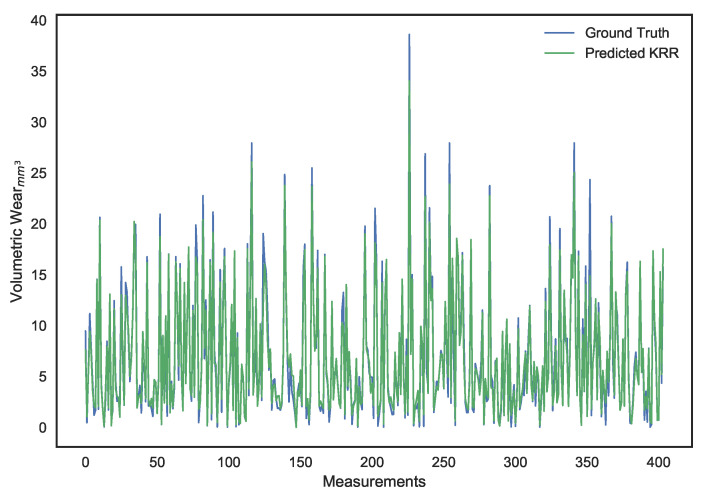
Surface wear prediction on the training set using $\chi^{2}$ kernel ridge regression.

**Figure 15:**
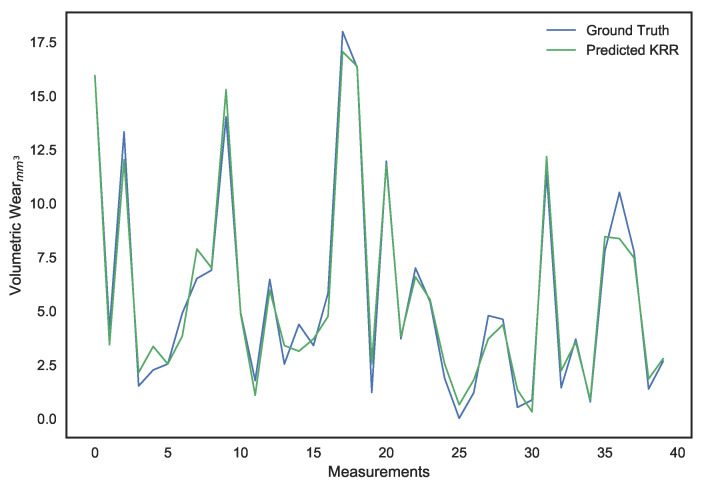
Surface wear prediction on the validation set using $\chi^{2}$ kernel ridge regression.

These parameters were set after hyper-parameter tuning. It can be noted that, while LS linear regression provides the lowest error on the training set, it is a result of overfitting since its performance on the validation set is dismal. Ridge regression provides better generalization and avoids the collinearity problem, but its performance isn’t ideal. The use of a $\chi^{2}$ kernel resulted in the validation error of ridge regression being nearly halved and a significantly higher $R^{2}$ score. The disparity in performance of the three models can be visually interpreted through the predictions shown in Figure 10, Figure 11, Figure 12, Figure 13, Figure 14 and Figure 15.

**Figure 16:**
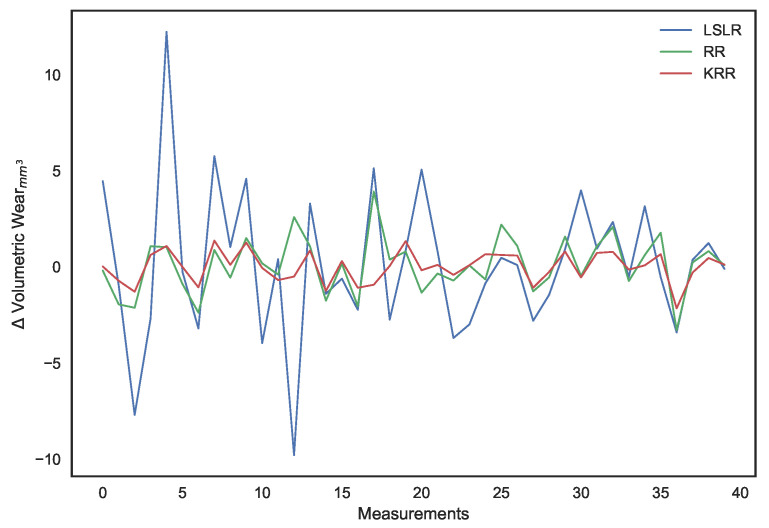
Deviation between model predictions and actual surface wear.

It can be observed that, apart from $\chi^{2}$ regression, the other models even predicted negative surface wear losses. The deviations of each of the model’s predictions from the actual volumetric loss can be noted in Figure 16, which illustrates how, as a whole, the deviations for the $\chi^{2}$ kernel ridge regression are smaller than other models. Most significantly, as demonstrated by the validation MAE metric, it predicts surface wear with an average deviation of under 1 $mm^{3}$

Since the load and lubrication conditions for each measurement were not factored into the input feature space for the prediction model, it is pertinent to ensure that the model is agnostic to these conditions and is not returning favorable performance for a subset of load and lubrication specifications. Additionally, it needs to be ensured that the performance of the $\chi^{2}$ kernel ridge regression model is generalized and not optimized only for a particular split of training and validation data split.

The dataset of 444 measurements was randomly sorted and split an additional nine times, while ensuring that there were a minimum of five measurements corresponding to each load and lubrication specification in a validation set. The KRR model, with the previously defined hyper-parameters, was trained and validated on these sets separately. The distribution of measurements against their specifications in the validation sets is provided in Table 4, with the breakdown of MAE achieved for different specifications tabulated in Table 5. The first set in the tables corresponds to the validation set used to compare KRR with other regression models.

Given the distribution of measurements against their specifications in Table 4, it can be ascertained that the prediction model is indeed agnostic to the load and lubrication conditions. This conclusion is backed up by the breakdown of metrics present in Table 5, from which it can be observed that the prediction error was below 1 $mm^{3}$ for nearly all cases. The tabulated MAE metrics also certify that the $\chi^{2}$ kernel ridge based prediction model is generalized. The overall averaged $R^{2}$ and MAE metrics are listed in Table 6 for the various training and validation sets. It can also be observed from the various validation sets that the surface wear was successfully predicted with an average disparity significantly under 1 $mm^{3}$ between the predicted volume and the ground truth.

## 4. Conclusions

In this work, the feasibility of predicting surface wear for a wear process on a pin on disc setup via airborne noise was studied. Qualitative signal analysis was performed on the recorded measurements in order to determine an appropriate methodology for feature extraction. The nature of the experiment and the manner in which wear was recorded were taken into account, and the task was approached from a time series perspective to allow the wear prediction to be modeled as a standard regression problem.

Possible regression models were examined in order to engineer an optimal solution for the input feature space. Consequently, $\chi^{2}$ kernel ridge regression provided predictions for surface wear with an average accuracy of within 1 $mm^{3}$ for both the training and validation sets of measurements, while being agnostic to the load and lubrication specification of each measurement. Performance of the model was cross-validated using multiple training and validation sets. Hence, it can be asserted that a functional quantification between surface wear and airborne noise, via its PSD, has been obtained.

Extensive validation of this trained model on unseen tribopair setups, i.e., new load and lubrication conditions, needs to be ascertained and will be carried out in a future study.

## Figures and Tables

**Table 1:** Pin material.

Steel Grade	Hardness (HRC)	Chemical Constitution (wt%)					
		Carbon	Tungsten	Molybdenum	Chromium	Vanadium	Silicon
HS3-3-3	64	1.00	2.98	2.83	4.27	2.30	-

**Table 2:** Breakdown of experiments and measurements.

Specification	Number of Experiments	Number of Measurements
No lubrication, no load	9	108
Lubrication, no load	8	96
Lubrication, 4.91 N load	10	120
Lubrication, 9.81 N load	10	120

**Table 3:** Performance of regression models.

	Metric	$R^{2}$		MAE (mm${}^{3}$)	
Model		**Training Set**	**Validation Set**	**Training Set**	**Validation Set**
Least-Squares		0.9759	0.3599	0.720	2.727
Ridge		0.9170	0.9034	1.284	1.173
Kernel Ridge		0.9600	0.9716	0.824	0.635

**Table 4:** Distribution of measurements in validation sets.

Specifications	Sets									
	1	2	3	4	5	6	7	8	9	10
No lubrication, no load	7	7	9	11	11	8	7	12	9	9
Lubrication, no load	10	14	5	10	11	9	10	8	13	12
Lubrication, 4.91 N load	13	8	13	6	12	11	8	10	9	8
Lubrication, 9.81 N load	10	11	13	13	6	12	15	10	9	11

**Table 5:** Breakdown of metrics across validation sets.

Specifications	MAE (${mm}^{3}$)									
	1	2	3	4	5	6	7	8	9	10
No lubrication, no load	0.690	1.007	0.705	0.742	0.772	0.811	0.424	0.991	0.979	0.764
Lubrication, no load	0.534	0.740	0.766	0.676	0.723	0.776	0.793	0.816	0.660	0.978
Lubrication, 4.91 N load	0.740	0.752	1.030	0.762	0.962	0.570	0.908	0.736	0.845	0.863
Lubrication, 9.81 N load	0.561	0.845	0.647	0.586	0.436	0.868	0.869	0.642	0.611	0.441

**Table 6:** Performance of KRR model on different training and validation sets.

	Metric	$R^{2}$		MAE (mm${}^{3}$)	
Model		**Training Set**	**Validation Set**	**Training Set**	**Validation Set**
1		0.960	0.972	0.824	0.635
2		0.960	0.952	0.814	0.817
3		0.960	0.954	0.821	0.799
4		0.960	0.956	0.827	0.678
5		0.960	0.945	0.824	0.765
6		0.960	0.957	0.819	0.752
7		0.960	0.957	0.820	0.780
8		0.958	0.962	0.834	0.805
9		0.960	0.945	0.820	0.763
10		0.959	0.967	0.828	0.759
Average		0.960	0.957	0.823	0.756

## Data Availability

Not applicable.

## Nomenclature

β	penalty term in ridge regression
w	weight vector in least-squares linear regression
X	matrix of input feature space for the measurements
y	vector of target variable in regression problem
γ	kernel variance
κ	kernel function
μ	mean of a signal
${\tilde{C}}_{XX}\left(e^{jw}\right)$	power spectral density estimate
*D*	number of segments
*d*	width of wear track
*E*	expectation operator
*h*	time horizon
$I_{WX,WX}^{M}\left(e^{jw},k\right)$	periodogram for segment of length *M* from signal *X*
*K*	number of segments
*M*	segment length of a signal, used in the Welch Method
*N*	total length of the signal
*R*	radius of wear track
*r*	pin end radius
$R_{xx}$	autocorrelation sequence of signal *X*
*V*	volumetric wear of disc in ${\mathrm{mm}}^{3}$
$w\left(n\right)$	window function
$X_{h}$	Spectral features for the noise measurement at horizon *h*
$y_{h}$	surface wear measured at horizon *h*
*Z*	normalization factor
